# The mediating role of college student psychological resilience in the relationship between wisdom and psychotic-like experiences

**DOI:** 10.3389/fpsyg.2026.1814754

**Published:** 2026-05-19

**Authors:** Han Wang, Yupu Zhu, Yan Gao, Fangfang Xu, Wei Su, Weiyan Zhao, Yayi Zong, Aoxue Zhang, Yan Wang, Min Chen

**Affiliations:** School of Mental Health, Jining Medical University, Jining, Shandong, China

**Keywords:** college students, mental health, psychological resilience, psychotic-like experiences, wisdom

## Abstract

Wisdom and psychological resilience function as critical adaptive mechanisms that buffer against mounting academic stressors and attenuate the risk of psychotic-like experiences (PLEs). Despite their theoretical importance, empirical inquiry into the interplay among wisdom, resilience, and PLEs within the undergraduate population remains underexplored. Therefore, the present study explores the mediating role of psychological resilience between wisdom and psychotic-like experiences to provide a theoretical basis for promoting the mental health of college students. A total of 1,974 college students from a selected medical college were included in the data analysis using a convenience sampling method. All participants completed three scales: the San Diego wisdom scale (SD-WISE), Community Assessment of Psychic Experiences (CAPE-P15), and the Connor-Davidson Resilience Scale (CD-RISC-10). And participants’ sociodemographic characteristics were collected. Mediation analysis indicated that psychological resilience mediates the association between wisdom and CAPE-P15 (a = 0.28, b = − 0.39, c = 0.08, c′ = 0.19). Corroborated by cluster analysis and network analysis, the findings suggest that resilience significantly buffers against the potential adverse impact of wisdom.

## Introduction

Generally, wisdom is defined as “the capacity of judging rightly in matters relating to life and conduct; soundness of judgement in the choice of means and ends; sometimes less strictly, sound sense, especially in practical affairs” (Birren and Svensson, 2005). A consistent view holds that wisdom refers to the ability to utilize existing psychological resources to address real-world challenges (Glück and Scherpf, 2022). Intelligence, by contrast, is a necessary but insufficient condition for wisdom. Wisdom research encompasses two major meta-theoretical communities (Dong et al., 2023), which differ in their conceptualization of wisdom and corresponding measurement approaches. On the one hand, the Berlin group defines wisdom from a cognitive perspective as cognitive processes and their products (Baltes and Smith, 1990), establishing the Berlin Wisdom Paradigm and later supplementing it with the Bremen Paradigm, On the other hand, other researchers conceptualize wisdom as relatively stable personal characteristics integrating cognition, motivation, emotion, and behavior. The self-evaluative SD-WISE scale, which assesses a suite of such wisdom-related traits, is a typical measurement tool for this approach. Despite their differences in focusing on “cognitive processes” versus “stable traits,” both paradigms provide valuable frameworks for understanding the nature of wisdom and guiding subsequent research.

This study adopts the trait-based perspective, as it allows for a direct examination of how individual differences in wisdom-related attributes relate to other psychological constructs. Central to the wisdom-mental health nexus is the regulatory function of specific adaptive components, among which emotional regulation and stress buffering are the core mechanisms. By leveraging enhanced emotional regulation, for instance, wise individuals are better equipped to modulate affective reactions to adversity, promoting positive daily functioning while mitigating psychological distress. Such mechanisms underpin observed associations between wisdom and improved well-being, as well as better physical and mental health outcomes, as well as better physical and mental health outcomes, with evidence further supporting that wisdom-related attributes are malleable through psychosocial interventions (Lee et al., 2020). Critically, wisdom’s role in mitigating the adverse effects of psychosocial stress on mental health (Ardelt and Jeste, 2018) provides a key link to subclinical psychological vulnerabilities—specifically, psychotic-like experiences (PLEs). In the context of the present study, PLEs are conceptualized as the most important outcome variable related to wisdom for two compelling reasons. First, there is a profound mechanistic alignment: PLEs are fundamentally characterized by cognitive biases and emotional dysregulation, which correspond exactly to the specific deficits that wisdom—defined by superior cognitive integration and emotional regulation—is uniquely equipped to counteract. Second, while prior research has broadly linked wisdom to general well-being, focusing on PLEs tests the protective capacity of wisdom against severe subclinical precursors to psychiatric disorders, moving beyond general distress. PLEs are often triggered by unregulated stress; therefore, wisdom can directly mitigate these key triggers by enhancing emotional regulation, reducing both the occurrence and associated distress of PLEs.

Psychotic experiences, sometimes called “psychotic-like experiences” (PLEs), can be categorized as positive PLEs (e.g., perceptual abnormalities, delusional thoughts) and negative PLEs (e.g., social withdrawal, lack of social pleasure). In the general population, the median prevalence rate is around 7–8% (Linscott and van Os, 2013). However, in undergraduates and youth, the prevalence has reached a higher number of 20.9%, strongly highlighting the emergency of preventing PLEs adolescents. Although most PLEs are transient, persistent ones may increase the risk of developing a psychotic disorder in the future (Lu et al., 2020). Cognitive biases play an important role in the development and maintenance of PLEs (Livet et al., 2020). Furthermore, the ability to regulate emotions, a crucial components of wisdom is a key factor influencing PLEs—related distress. Adaptive strategies may reduce distress levels, providing a theoretical basis for early psychological interventions targeting high-risk groups (Osborne et al., 2017). Preliminary investigations reveal that wisdom reduces the occurrence and distress of PLEs (Wu et al., 2022). However, the specific underlying mechanism through which wisdom alleviate PLEs symptoms require further clarification.

Demographically, females tend to score higher on the SD-WISE dimensions of self-reflection, acceptance of different points of view, pro-social behavior, and social advice, while males score higher on emotion regulation and decisiveness (Treichler et al., 2022). These differences may be due to biological and sociocultural factors. Additionally, studies suggest that wisdom may increase with age: in complex decision-making relying on long-term contexts, older adults perform better by virtue of their ability to integrate experience and reason prospectively (Worthy et al., 2011). Thus, wisdom is a key factor in maintaining mental health. Enhancing wisdom levels can be an effective way to increase psychological resilience, helping people better cope with challenges in different life stages.

Psychological resilience refers to the ability to fare better than expected in the face of adversity, characterized by minimal disruptions or even improvements in psychological functioning. It is context-dependent and emerges through adaptive affect-regulation processes that mitigate the impact of stress and negative emotions over time (Troy et al., 2023). According to the psychological resilience intrgrative model, resilience activates internal protective factors to counteract a negative environment (Chen et al., 2022). It core protective mechanism is two aspects: it helps individuals maintain their baseline psychological health; and it promotes the effective use of internal and external resources to adapt to the environment. Consequently, highly resilient individuals can not only resist the unfavorable situations but also actively use supportive resources to significantly reduce negative emotions maintain psychological balance (Davydov et al., 2010; Ong et al., 2009).

Students are influenced by a variety of stress sources that can deteriorate their mental health (Mao et al., 2019). Using self-report data, this study investigates the relationships among wisdom, psychological resilience, and positive PLEs in college students, with a particular focus on the mediating role of psychological resilience. Based on the research objectives, the following two hypotheses are proposed: Hypothesis 1 suggests that wisdom is positively correlated with psychological resilience, but negatively correlated with positive PLEs. Hypothesis 2 posits that psychological resilience plays a partial masking role in the relationship between wisdom and positive PLEs, meaning that high psychological resilience can mitigate the potential mental health risks associated with high levels of wisdom. By integrating data from relevant questionnaires, this study is the first to systematically explore the complex relationships among wisdom, psychological resilience, and PLEs in a college student population, providing new empirical evidence for understanding the multifaceted impact of wisdom on mental health.

## Methods

This was a cross-sectional observational study using self-report measures. *A priori* power analysis using G*Power 3.1 indicated that a total sample size of *N* = 314 was required to detect a medium correlation (*r* = 0.2) with *α* = 0.05 (two-tailed) and power = 0.95. This study adopted convenience sampling to recruit undergraduate students from Jining Medical University, with data collection conducted between 2019 and 2021. Online questionnaires were designed and distributed through the Chinese online survey platform “Questionnaire Star” (www.wjx.com, also known as Wenjuanxing), with school administrative staff assisting in distributing the questionnaires via social media channels to reach potential participants. This research distributed a total of 1,974 questionnaires, and 1,240 undergraduates initially participated in the survey. To ensure data quality, two exclusion criteria were set: first, using the interquartile range method, participants who completed the questionnaire in an extremely short time (less than 207 s) were excluded, as this might indicate inattentive responses, resulting in the exclusion of 62 participants; second, for participants who self-reported having mental illnesses (25 in total), since the study did not collect clinical diagnostic data, their responses remained relevant to the research objectives, so this group was not excluded. We did not exclude the participants who self-reported a history of mental illness. This is because the researchers were unable to determine whether this self-reported medical history was based on the self-diagnosis of participants or professional psychiatric diagnosis. Therefore, we decided not to rely on this self-report. Meanwhile, we also verified the correlation diagnosis after excluding these patients. The results showed no difference in significance. Moreover, 2 participants were also excluded due to their failure to provide detailed age and gender data. Moreover, 2 participants were also excluded due to their failure to provide detailed age and gender data. After the above screening, 1,176 participants were finally included in the data analysis; these data were used for subsequent descriptive analysis.

### Sample size justification

Although a convenience sampling approach was utilized, the final sample size of 1,176 valid participants was rigorously evaluated for statistical adequacy based on the requirements of our primary analytical models. For the multiple linear regression model, the sample size vastly exceeds the conventional rule of thumb, which recommends 10 to 20 observations per predictor variable to maintain adequate statistical power. Furthermore, for the psychological network analysis, a large sample is imperative to ensure the stability of edge estimation. Based on recent literature criteria indicating that 22.1 to 55.5 observation samples per edge yield robust evidence (Huth et al., 2025), our network composed of 25 edges provides an average of 47.04 samples per edge. Therefore, the obtained sample size is exceptionally sufficient to guarantee the reliability and stability of both the regression and network topology analyses in the present study.

### Research design

This study aims to explore the impact of wisdom on the mental health of the college students. Therefore, we first conduct an exploratory analysis of the correlations among CAPE–P15, SD–WISE, and CD–RISC–10, hoping to clarify their mutual influences. Since wisdom is manifested as the subjects’ ability to handle real–life problems, and this ability may interfere with the subjects’ mental health, we choose to use SD–WISE and CD–RISC–10 as independent variables and CAPE - P15 as the dependent variable. On the one hand, we verify the collinearity among the variables to provide a basis for the mediation analysis. On the other hand, we also attempt to use the predictive power of SD–WISE and CD–RISC–10 for CAPE–P15 to explain the influence of wisdom, psychological resilience, and the subjects’ behavioral abilities on mental health.

Meanwhile, based on the above - mentioned research, the researchers constructed a mediation model to further elaborate the mutual influences among the three variables. The results revealed a complex mediating relationship among them. Finally, k - mean cluster analysis was used to further verify the conclusions of the above - mentioned analysis. Combining with partial correlation network analysis, the researchers further attempted to explain the possible reasons for the complex effects of wisdom on mental health.

### Ethics approval

This study was conducted in strict accordance with the Declaration of Helsinki and was formally approved by the Ethics Committee of Jining Medical University (Protocol Number: [2016/036]). All participants signed informed consent forms electronically prior to commencing the survey.

### Measurements

#### Demographic information and psychiatric history

The demographic profile in this study encompassed a total of 11 items. Regarding the demographic information of the participants, in addition to collecting their age, gender, and ethnicity, the researchers also gathered data on their family situations, including whether they were only children, whether their parents were divorced, whether they were from single-parent families, whether they had experience as left-behind children, and the educational attainment of their parents. The researchers also collected information on their place of origin (the province where they attended high school before entering university), self-reported past psychiatric history, and family history of mental health.

### Community assessment of psychotic experiences–positive 15-items scale (CAPE-P15)

Derived from the full CAPE questionnaire, the CAPE-P15 is primarily used for self-screening of subclinical and above-threshold psychiatric symptoms in non-clinical populations, demonstrating good reliability and validity among youth (Capra et al., 2013). It comprises three subscales: Persecutory Ideation (PI), Perceptual Abnormality (PA), and Bizarre Experiences (BE) (Villacura-Herrera et al., 2024). Using a 4-point Likert scale (0 = “completely disagree,” 3 = “completely agree”), the total score ranges from 0 to 45, with higher scores indicating more frequent and severe positive psychotic-like experiences (range = 0–3). Previous studies have confirmed the CAPE-P15 as a valid and reliable tool for assessing psychiatric experiences in college students, with high internal consistency across all subscales (Sun et al., 2020).

### San Diego wisdom scale (SD-WISE)

The SD-WISE, developed by Thomas et al. as a shortened version of the WISE scale, has a Chinese adaptation validated by Wu et al. (2023) from the Second Xiangya Hospital of Central South University. Focusing on general domains of wisdom, it assesses six dimensions: social advising, emotional regulation, prosocial behaviors, insight, tolerance for divergent values, and decisiveness. The scale consists of 24 questions divided into six dimensions, each with four questions, with higher scores corresponding to higher levels of wisdom (range = 1–5) (Thomas et al., 2019).

### Connor-Davidson resilience scale-10 (CD-RISC-10)

Psychological resilience was measured utilizing the Chinese version of the 10-item Connor-Davidson Resilience Scale (CD-RISC-10) (Ye et al., 2018). The original CD-RISC, revised in 2003, evaluates an individual’s ability to cope successfully and adapt to adversity, encompassing five factors: personal competence, tolerance for negativity, acceptance of change, control, and spirituality (Development of a new resilience scale: the CD-RISC). The CD-RISC-10 is a 10-item shortened version using a 5-point Likert scale (0 = “never,” 4 = “always”), with total scores ranging from 0 to 40 (range = 0–4); higher scores indicate greater psychological resilience. Previous research has shown a negative correlation between high resilience and mental disorders (Scali et al., 2012). The CD-RISC-10 demonstrates reliable psychometric properties in college student populations, and the Chinese version used in this study showed good internal consistency.

### Statistical analysis

All statistical calculations were completed by writing scripts in Python, and the scripts used for statistical calculations have been open–sourced^1^. Statistical significance was defined as *p* < 0.05. To systematically explore the complex relationships among wisdom, resilience, and PLEs, we employed an integrated analytical framework. This framework sequentially utilized: (1) mediation analysis to test the path from wisdom to PLEs via resilience; (2) K-means cluster analysis to identify participant subgroups; and (3) psychological network analysis to reveal the interactive structure of the constructs.”

### Partial correlation analyses

Researchers conducted partial correlation analyses on the three questionnaires to obtain the correlations among them. When conducting partial correlation analysis, demographic data including age, gender, nationality, family income, and interpersonal relationships were used as covariates for regression. This was done to prevent statistical heterogeneity arising from population differences. For specific variables, please refer to Table 1. All partial correlation results were finally obtained after FDR correction.

**Table 1:** Demographical variables (*N* = 1,176).

Variables	Category/unit	*n*	%	Mean± SD
Age		1,176		19.56 ± 1.33
Gender	Female	696	58.18	
	Male	480	40.82	
Nationality	Han	1,145	97.36	
	Ethnic minorities	31	2.64	
Fixed boyfriend or girlfriend	Yes	266		
	No	910		
Single parent family	Yes	99	8.42	
	No	1,077	91.58	
Only child	Yes	462	39.29	
	No	714	60.17	
Left behind children	Yes	197	16.75	
	No	979	83.25	
Family’s economic situation	Extremely difficult	16	1.36	
	Very difficult	61	5.19	
	Somewhat difficult	224	19.05	
	Similar to most people	817	69.47	
	Slightly wealthy	58	4.93	
	Very wealthy	0	0	

*n* = frequency, % = percentage.

### Multiple linear regression equation model

Considering the partial correlation among CAPE-P15, SD-WISE, and CD-RISC-10, the researchers are concerned about the impacts of SD-WISE and CD-RISC-10 on the mental state of the participants. Therefore, the investigators took the scores of these two questionnaires as predictors and used the forced-entry method to fit a multiple linear regression equation model, aiming to predict the changes in CAPE-P15 scores. Meanwhile, the researchers conducted a collinearity diagnosis on the predictors (Table 2) to ensure the accuracy of the model.

**Table 2:** Multiple linear regression model.

Variables	Unstandardized coefficients		*t*	*p*	95% confidence interval		Collinearity statistics	
	*β*	Std err			[0.025]	[0.975]	Tolerance	VIF
Constant	4.576	0.923	38.178	0.000	4.319	4.787		
SD-WISE	0.081	0.012	6.702	0.000	0.589	1.077	0.920	1.087
CD-RISC-10	−0.222	0.016	−13.995	0.000	−1.984	−1.496	0.920	1.087

std err = standard error, VIF = variance inflation factor, β = standardized regression coefficient, t = t statistic, *p* = *p*-value.

### Mediation model

To further depict the relationships among CAPE-P15, SD-WISE, and CD-RISC-10, the researchers constructed a mediation model among the three questionnaires. The construction method of the mediation model refers to the published statistical methods (Baron and Kenny, 1986; Fairchild and McDaniel, 2017). In the model, the investigators set CD-RISC-10 as the mediating variable in the relationship between the independent variable SD-WISE and the dependent variable CAPE-P15. When constructing the model, the researchers first examined whether the independent variable affected the dependent variable. In the second step, the investigators calculated the effect relationship between the independent variable and the hypothesized mediating variable. In the third step, after controlling for the independent variable, the researchers analyzed the relationship between the hypothesized mediating variable and the dependent variable. In the fourth step, the investigators tested for the partial mediation effect, that is, whether the effect between the independent variable and the dependent variable was weakened. If the analysis results meet the following conditions: the tests in the second and third steps are statistically significant, and the effect of the independent variable in the fourth step is significantly reduced.

### K–means cluster analysis

The investigators conducted a cluster analysis on the participant group using the k-mean clustering method. Prior to the cluster analysis, the researchers first used the elbow method to determine that the optimal number of clusters was 3 (Supplementary Figure 1). Then, the investigators performed the clustering using the total scores of SD-WISE and CD-RISE-10. The preliminary clustering results showed that the number of participants in cluster 3 was less than 3% of the total number of participants (*n* = 31, accounting for 2.64%, Supplementary Figure 2), In addition, when *k* = 4, the proportion of Cluster 3 was still no more than 3% (*n* = 31, accounting for 2.64%, Supplementary Figure 3). Moreover, one way ANOVA was performed on the CAPE - P15 scores of all subjects based on the clustered groups (Supplementary Figure 4). The results suggested that the final conclusion did not change significantly. Therefore, the researchers adjusted the clustering strategy and re-conducted the clustering with *k* = 2. Finally, based on the clustering results, the investigators assigned labels to the participants and divided them into two groups. An independent samples *T*-test was performed on the CAPE-P15 scores between the two groups to verify whether the classification results could predict the stratification of CAPE-P15 scores.

### Psychological network analysis

Based on the partial correlation matrix obtained from the above analysis, a psychological network model was constructed. The researchers defined the sub-items of the three questionnaires, namely CAPE-P15, CD-RISC-10, and SD-WISE, as nodes, and the partial correlation coefficients between the nodes as edges. A *post hoc* power analysis was conducted using G*Power 3.1 for the bivariate correlation test. With a sample size of *N* = 1,176, a two-tailed *α* level of 0.05, and an assumed population correlation of *ρ* = 0.707, the achieved power (1-*β*) was 1.00, indicating that the study had sufficient statistical power to detect the hypothesized strong correlation with a near-zero probability of Type II error. Therefore, the researchers included correlations with |*r*| > 0.2 as edges in the network analysis. After filtering out the weak associations, an undirected graph was constructed using graph theory and network analysis. In the network topology analysis, the researchers calculated degree centrality, betweenness centrality, and closeness centrality. Furthermore, community detection was carried out using the Louvain module word (Blondel et al., 2008) to measure the relationships between nodes.

## Results

### General descriptive statistics

In this study, a total of 1,974 participants were recruited, and 1,176 participants were included in the final data analysis. The average age of all participants was 19.56 ± 1.33 (mean ± SD), among whom 696 were female and 480 were male. Other study variables are presented in the variable in Tables 1, 3. Meanwhile, the Cronbach’s Alpha coefficient was used to examine the internal consistency reliability of the SD-WISE, CAPE-P15, and CD-RISC-10 in the sample of this study. The results showed that the Alpha coefficient of the SD-WISE was 0.83, that of the CPAE-P15 was 0.83, and that of the CD-RISC-10 was 0.96. All of these values were higher than the recommended standard of 0.80, indicating that these scales had extremely high reliability for the sample of this study.

**Table 3:** General descriptive statistics of questionnaires.

Questionnaires	Mean ± SD	Range
SD-WISE		
Total	77.41 ± 10.30	120–24
Decisiveness	11.93 ± 2.44	20–4
Emotional regulation	12.61 ± 1.97	20–4
Social advising	12.09 ± 1.89	20–4
Prosocial behaviors	12.53 ± 2.17	20–4
Insight	13.00 ± 2.29	20–4
Tolerance for divergent values	15.26 ± 2.87	20–4
CAPE-P15		
Total	4.55 ± 4.43	26–0
Persecutory ideation	2.30 ± 1.97	11–0
Bizarre experiences	1.91 ± 2.31	14–0
Perceptual abnormalities	0.35 ± 0.91	6–0
CD-RISC-10	28.33 ± 7.84	40–0

### Partial correlation analysis

**Figure 1:**
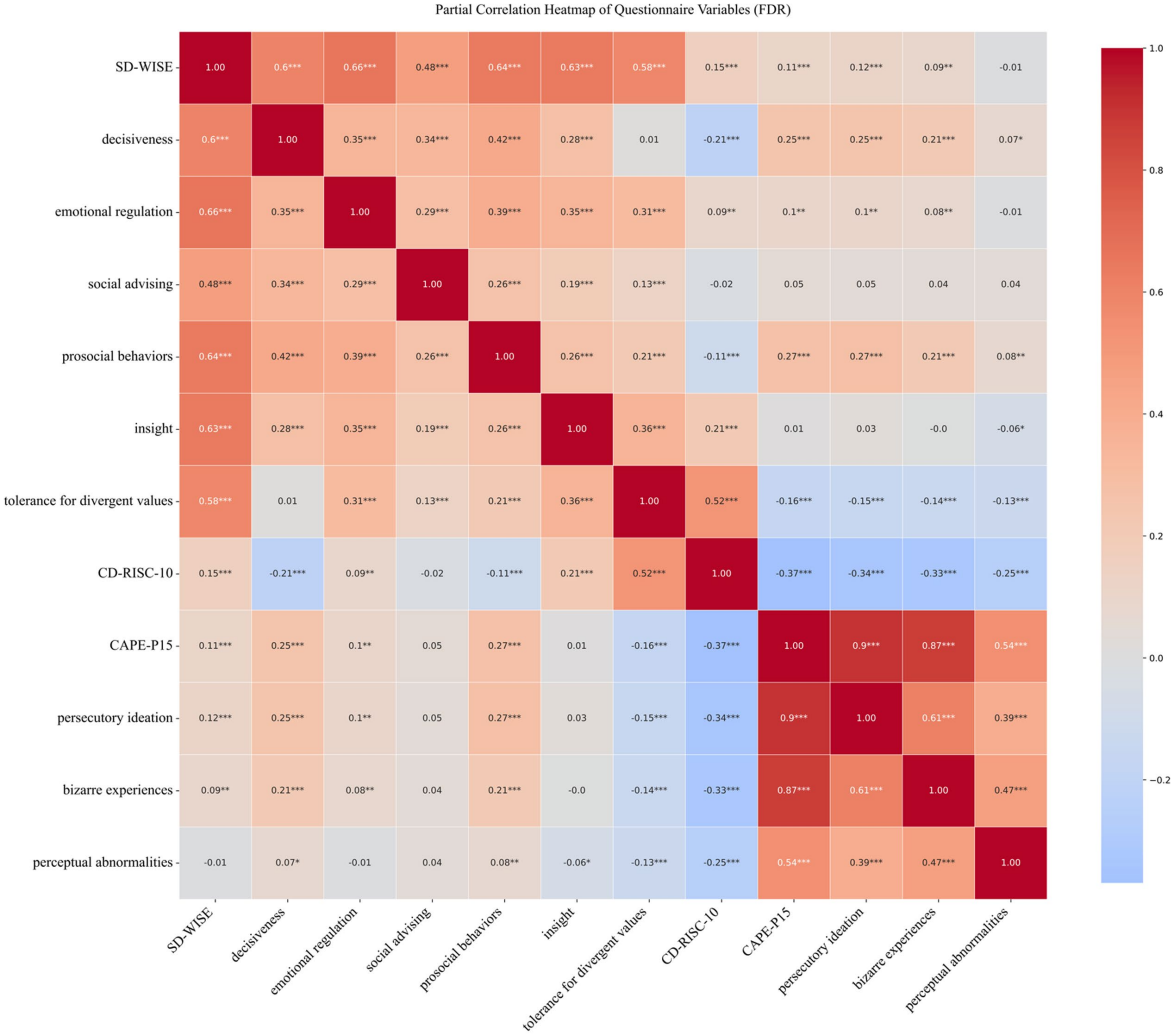
Partial correlation heatmap of questionnaire variables (FDR). The numbers in the heatmap are correlation coefficients. Warm colors represent positive correlations, while cool colors represent negative correlations. The larger the absolute value of the correlation coefficient, the darker the color. *p < 0.05, ** *p* < 0.01, *** *p* < 0.001.

The investigators conducted a correlation analysis of SD-WISE using CAPE-P15 and CD-RISC-10 (Figure 1). The results showed that there was a significant positive correlation between the total score of SD-WISE and the total score of CD-RISC-10 (*r* = 0.15, *p* < 0.001). There was a negative correlation between the total score of CAPE-P15 and the total score of CD-RISC-10 (*r* = −0.37, *p* < 0.001), and a significant positive correlation between the total score of SD-WISE and the total score of CAPE-P15 (*r* = 0.11, *p* < 0.001).

Among the sub-item scores of the questionnaires, there was a positive correlation between “tolerance for divergent values” and CD-RISC-10 (*r* = 0.52, *p* < 0.001). Regarding the sub-item scores between SD-WISE and CAPE-P15, there was a positive correlation between “prosocial behaviors” and “persecutory ideation” (*r* = 0.27, *p* < 0.001). There were positive correlations between “decisiveness” and “persecutory ideation” (*r* = 0.25, *p* < 0.001), as well as between “decisiveness” and “bizarre experiences” (*r* = 0.21, *p* < 0.001). Also, there were positive correlations between “prosocial behaviors” and “persecutory ideation” (*r* = 0.27, *p* < 0.001), and between “prosocial behaviors” and “bizarre experiences” (*r* = 0.08, *p* < 0.001).

### Multiple linear regression model

Before establishing the multiple linear regression model with CAPE-P15 as the dependent variable, we performed a multicollinearity diagnosis on all predictors. The results indicated that the collinearity among the predictors was weak. The model could significantly predict the total CAPE-P15 score (*R*^2^adj = 0.147, *F* = 44.912, *p* < 0.001). Notably, the adjusted *R*^2^adj of 0.147 indicates that the combined predictors account for 14.7% of the total variance in positive psychotic-like experiences, demonstrating moderate explanatory power. Examining the standardized coefficients (*β*) to evaluate the relative effect sizes, we found that psychological resilience exerts a substantial negative predictive effect (CD-RISC-10: *β*adj = −0.393, *t* = −13.995, *p* < 0.001), whereas wisdom shows a significant but relatively smaller positive predictive effect (SD-WISE: *β*adj = 0.188, *t* = 6.702, *p* < 0.001) within the model.

### Mediation model

**Figure 2:**
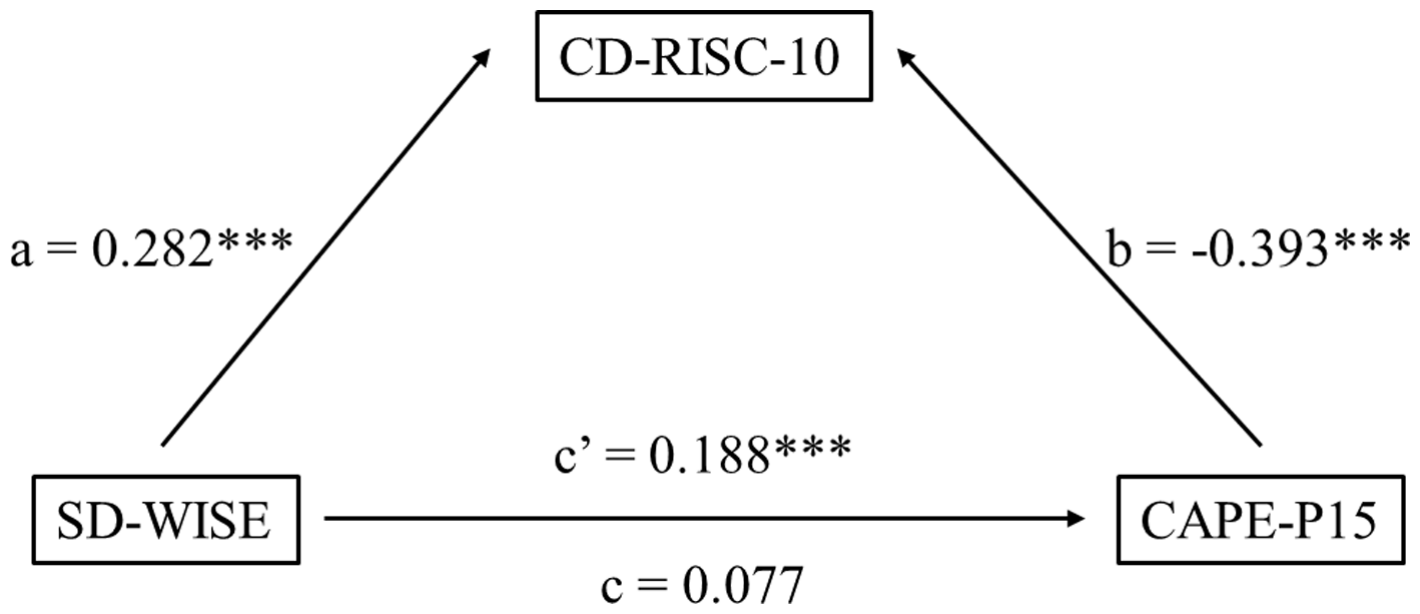
Mediation model. The model of the relationship between SD-WISE and CAPE-P15. (a–c, c’) The standardized *β* coefficient for each path. *p < 0.05, **p < 0.01, ***p < 0.001.

Researchers established a mediation model (Figure 2) with SD-WISE as the independent variable, CAPE-P15 as the dependent variable, and CD-RISC-10 as the mediating variable. The results showed that the total effect of SD-WISE on CAPE-P15 was 0.077 (*p* = 0.059, SE = 0.039, 95% CI [−0.002, 0.148]). The direct effect, after controlling for CD-RISC-10, was 0.188 (*p* < 0.001, SE = 0.044, 95% CI [0.101, 0.272]). Additionally, the effect of SD-WISE on CD-RISC-10 was 0.282 (*p* < 0.001, SE = 0.054, 95% CI [0.168, 0.380]). The effect of CAPE-P15 on CD-RISC-10 was −0.393 (*p* < 0.001, SE = 0.029, 95% CI [−0.451, −0.336]). The results of the mediation model suggested that CD-RISC-10 had a masking effect on the correlation between SD-WISE and CAPE-P15. Meanwhile, in terms of mathematical techniques, the fact that a and b have opposite signs is recognized as the existence of a competitive mediation. Through mathematical reasoning, it was found that the total effect test is not beneficial in the mediation model. Therefore, the researchers believe that this mediation model holds (Jiang et al., 2021). Researchers believe that the inverse correlation between a and b is an indication of a masking effect (Chen et al., 2022; Qin et al., 2025).

### Cluster analysis

**Figure 3:**
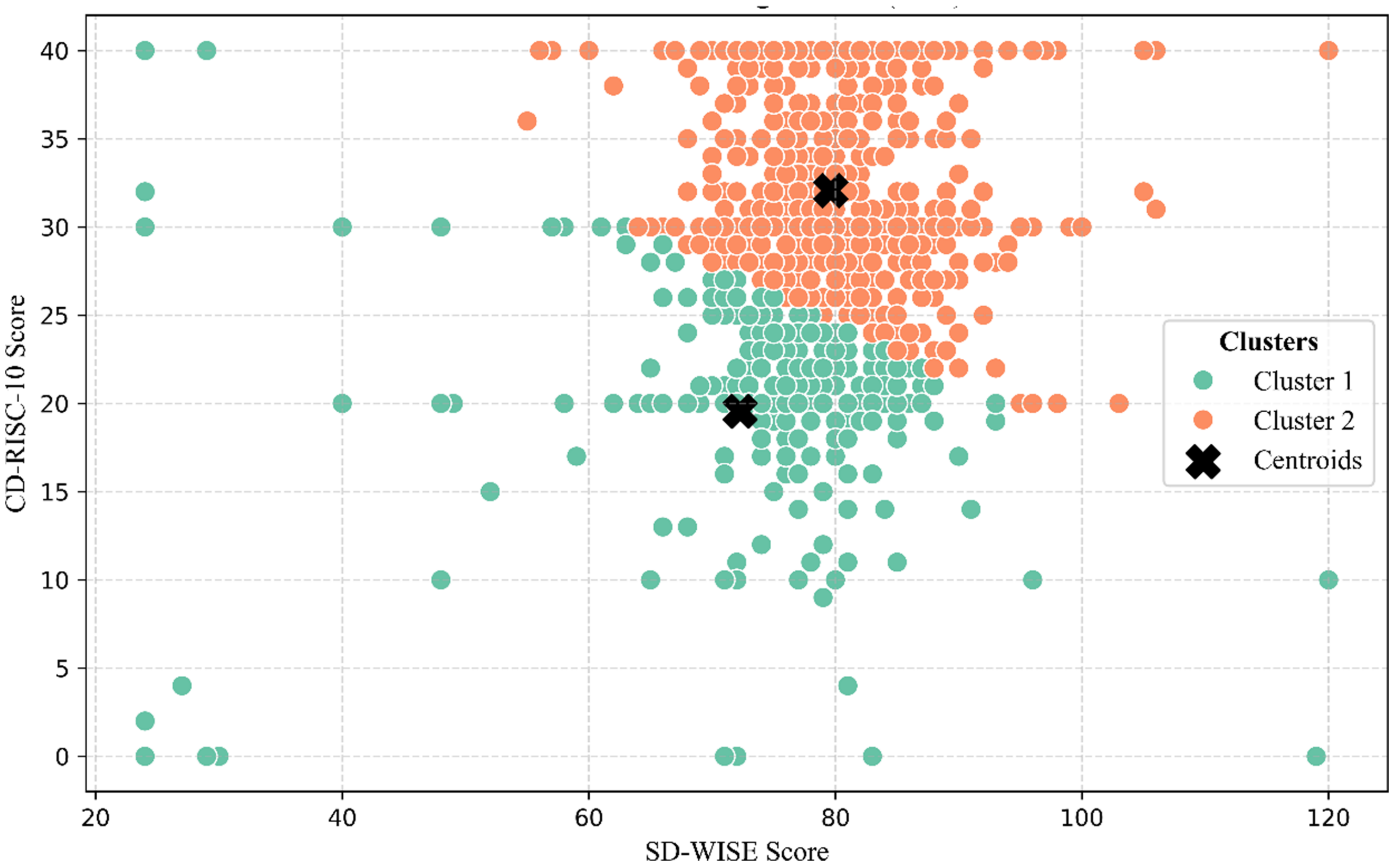
*K*-means clustering results (*K* = 2). The black cross marks indicate the cluster centroids. Green dots represent cluster 1, where both CD-RISC-10 and SD-WISE scores are low. Orange dots represent cluster 2, where both CD-RISC-10 and SD-WISE scores are high.

**Figure 4:**
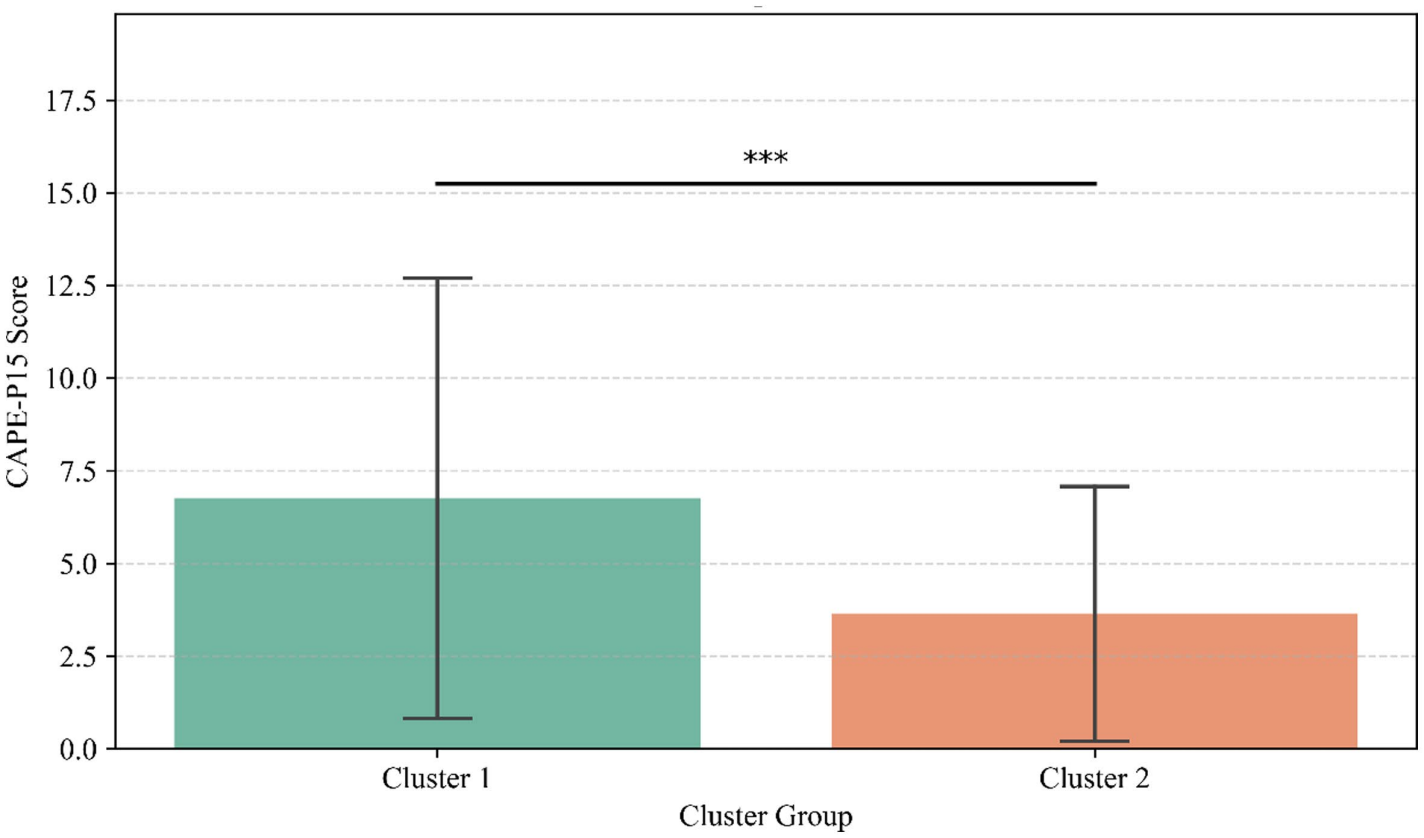
CAPE-P15 scores comparison between clusters. Results of the independent-samples *t*-test on CAPE-P15 scores between the two clusters after *k*-mean cluster analysis. ***p < 0.001.

Based on the finally determined k-value, the investigators employed the k-mean method to cluster all the subjects into two groups according to the scores of SD-WISE and CD-RISC-10 (Figure 3). The results indicated that 29.93% of the subjects (*n* = 352) could be classified into Cluster 1, and 70.07% of the subjects (*n* = 824) could be classified into Cluster 2. Subsequently, the investigators labeled all the subjects according to the clustering results and calculated the difference in CAPE-P15 scores between the two groups. The findings revealed a significant difference in CAPE-P15 scores between the two groups (*t* = 9.19, *p* < 0.001) (Figure 4). Thus, the investigators conclude that the scores of SD-WISE and CD-RISC-10 can predict the CAPE-P15 scores.

### Psychological network analysis

#### Network estimation and centrality measure analysis

**Figure 5:**
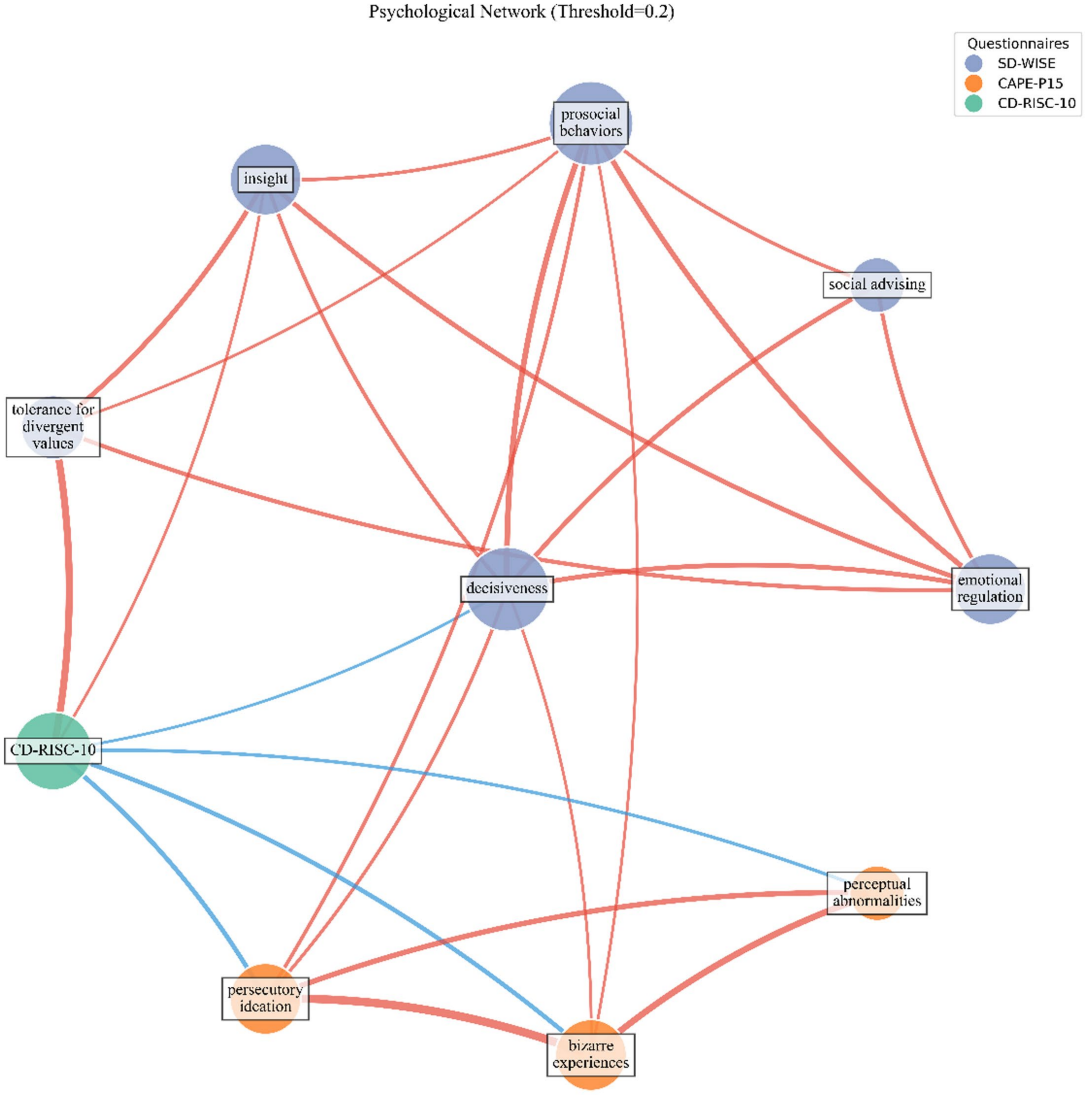
Psychological network. Psychological network between sub-items of wisdom (SD-WISE), psychological resilience (CD-RISC-10), and positive psychotic-like experiences (CAPE-P15). Red node: sub-items of SD-WISE. Green node: CD-RISC-10. Orange node: sub-items of CAPE-P15. Red lines represent a positive correlation between nodes, and blue lines represent a negative correlation between nodes. The larger the node, the greater its proportion in the network, and the thicker the line, the stronger the correlation between nodes.

In the topological network composed of 10 nodes and 25 edges, the network density is 0.56, the average clustering coefficient is 0.69, and the average shortest path is 1.49. Centrality measure analysis shows that “decisiveness,” “prosocial behaviors,” and “CD-RISC-10” are the key nodes in the network (Figure 5). Among them, “decisiveness” has the highest degree centrality, betweenness centrality, and closeness centrality.

**Figure 6:**
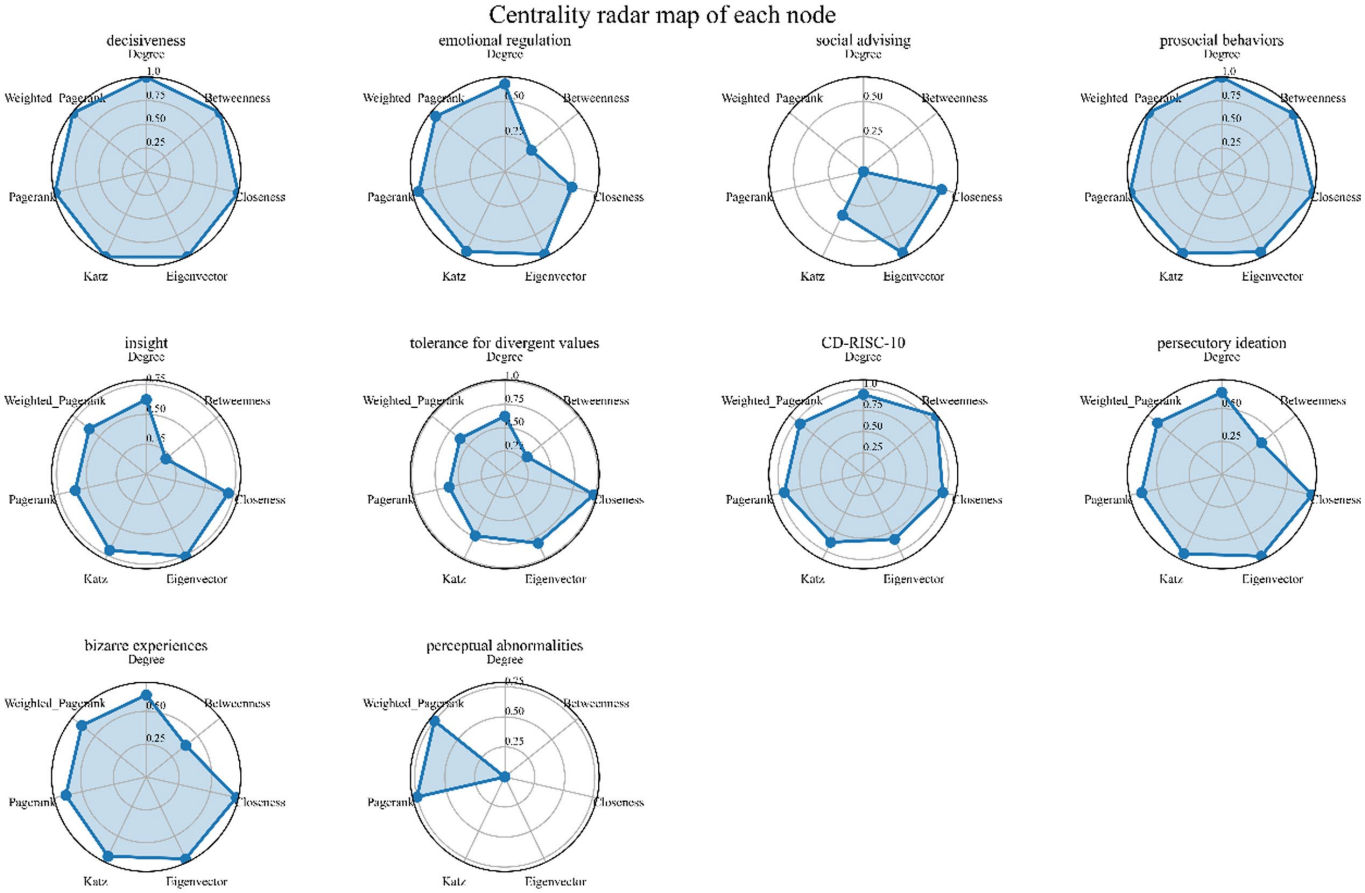
Centrality radar map of each node. There are multiple indicators used to measure the importance of nodes within a network, including: Degree: The degree of a node refers to the number of edges directly connected to it. Nodes with high degree centrality have higher connectivity within the network. Betweenness: The betweenness centrality of a node is the number of shortest paths passing through that node. Nodes with high betweenness centrality act as ‘bridges’ in the network, crucial for the flow of information or resources. Closeness: The closeness centrality of a node is the average length of the shortest paths from that node to all other nodes in the network. Nodes with high closeness centrality are typically located centrally within the network. Eigenvector: Eigenvector centrality takes into account the degrees of a node’s neighboring nodes. A node with high eigenvector centrality has neighbors with high centrality. Katz: Katz centrality is a weighted centrality measure based on path length, considering the importance of all possible paths between nodes. Pagerank: Originally used for webpage ranking, it measures the importance of nodes based on the quality and quantity of their links. Weighted Pagerank: This is a variant of the PageRank algorithm that incorporates the weights of edges, acknowledging that different links may have varying importance.

Regarding the connections between nodes (Figure 5), apart from the internal connections among the sub-items of the questionnaires, the correlation between “tolerance for divergent values” and psychological resilience (CD-RISC-10) is the strongest. Next is the correlation between “prosocial behaviors” and “persecutory ideation.” There are connections between wisdom “SD-WISE” and positive psychotic-like experiences “CAPE-P15,” between “decisiveness” and “persecutory ideation” as well as “bizarre experiences.” “Prosocial behaviors” has direct connections with “persecutory ideation” and “bizarre experiences.” Psychological resilience, which acts as a mediating variable between wisdom and positive psychotic-like experiences, links “tolerance for divergent value”, “insight”, and “decisiveness” to all sub-items of “CAPE-P15.” As a mediating variable between wisdom and positive psychotic-like experiences, psychological resilience also has relatively high betweenness centrality and closeness centrality (Figure 6). “Perceptual abnormalities” has a relatively weak overall importance in the network (Figure 6).

#### Network accuracy and stability

**Figure 7:**
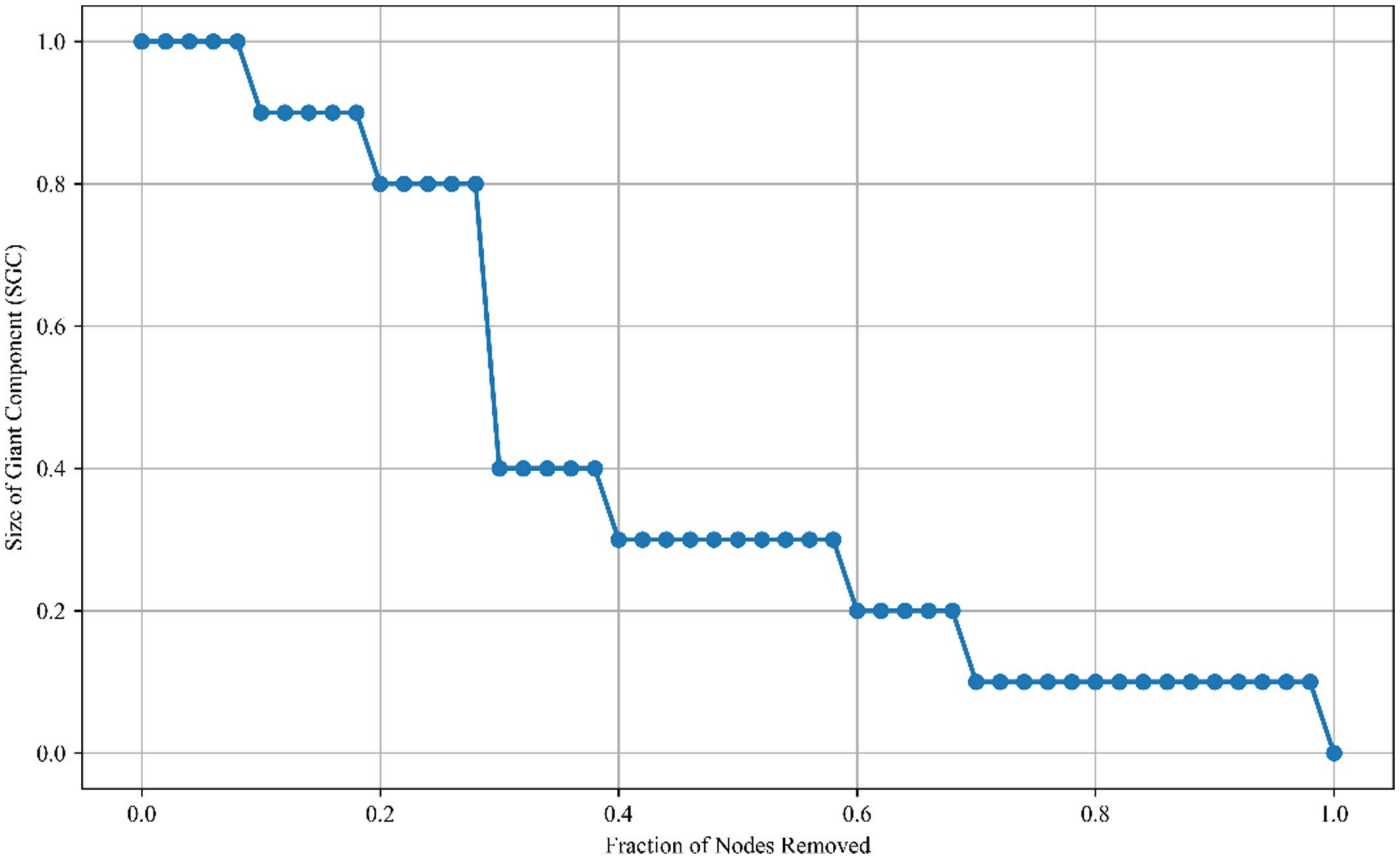
Network robustness to targeted attacks.

After 5,000 Bootstrap tests, the confidence intervals for the nodes, edges, and density of the network are all relatively small, demonstrating the high stability of the network. The retained rate of the size of giant component (SGC) was 30.0% after attacking 50% of the nodes, and 10.0% after attacking 80% of the nodes (Figure 7). The network’s robustness against targeted attacks did not decay linearly; there was a “critical threshold.” When the proportion of removed nodes exceeded specific values (such as intervals like 0.2 and 0.4), the size of the SGC dropped sharply, and the network rapidly shifted from “highly connected” to “fragmented.” This reflects the crucial role of hub nodes in the network structure, indicating that targeted attacks can efficiently destroy network connectivity if they are directed at core nodes.

## Discussion

This study explored the potential impacts of wisdom on mental health and investigated the reasons behind these impacts. The researchers measured the wisdom levels of healthy college students using the SD-WISE questionnaire from multiple dimensions, including insight, prosocial behavior, emotion regulation, decisiveness, self-reflection, and tolerance. Additionally, through the questionnaire, the researchers examined the psychological resilience and mental state of the participants. The results showed significant correlations among the wisdom levels, psychological resilience, and PLEs of the measured participants. Further construction of a mediation model revealed that psychological resilience played a masking role between wisdom levels and PLEs. The results of the network analysis suggested that the impact of wisdom on mental state might be multi-faceted, with prosocial behavior and insight being key bridging nodes.

### Summary of key findings

In this study, we explored the potential impacts of wisdom on mental health and investigated the underlying reasons for these impacts among healthy college students. Our integrated analytical framework revealed significant correlations among wisdom, psychological resilience, and PLEs. Specifically, partial correlation analysis demonstrated a positive relationship between wisdom (SD-WISE) and resilience (CD-RISC-10) (*r* = 0.15, *p* < 0.001), but also a positive relationship between wisdom and positive PLEs (CAPE-P15) (*r* = 0.11, *p* < 0.001). Furthermore, our mediation analysis identified the magnitude and nature of these relationships, revealing that psychological resilience exerts a partial masking effect on the relationship between wisdom levels and PLEs. Network analysis systematically elaborated on this dynamic, highlighting that specific sub-dimensions—namely prosocial behavior and decisiveness—serve as key bridging nodes.

### Interpretation aligned with hypotheses

These statistical findings directly address and expand upon our initial hypotheses. Consistent with Hypothesis 1, we observed a significant positive correlation between wisdom and psychological resilience (*r* = 0.15), indicating that higher levels of wisdom generally confer psychological health benefits by enhancing the ability to cope with stress. However, contrary to the simplistic expectation that wisdom would strictly correlate negatively with positive PLEs, our results explicitly revealed a significant positive correlation between the SD-WISE and CAPE-P15 total scores (*r* = 0.11). Addressing Hypothesis 2, our mediation model clarified the exact nature and magnitude of this masking effect. We found that the direct effect of wisdom on PLEs is significantly positive (c’ = 0.19, *p* < 0.001). However, wisdom also positively predicts psychological resilience (a = 0.28, *p* < 0.001), which in turn strongly negatively predicts PLEs (*b* = −0.39, *p* < 0.001). Because the indirect path through resilience acts in the opposite direction (negative) to the direct path, resilience partially masks the overall positive impact of wisdom on PLEs, suppressing the total effect (c = 0.08, *p* < 0.001). This mathematically demonstrates that an increase in psychological resilience can effectively buffer the potential negative mental health effects brought about by high wisdom. Our K-means cluster analysis provided a complementary, person-centered taxonomic perspective that further corroborated this masking interaction. The emergence of the “high wisdom-high resilience” and “low wisdom-low resilience” subgroups is not arbitrary; rather, these data-driven phenotypes align closely with theories of individual differences in cognitive-emotional regulation capacity. This person-centered approach complements our mediation model by demonstrating that the potential subclinical vulnerabilities introduced by high wisdom are safely absorbed and counterbalanced when the individual concurrently possesses a matched, high level of psychological resilience.

### Integration of the analytical framework

To comprehensively delineate the complex relationship between wisdom and psychotic-like experiences, our study employed a triangulated analytical framework. While the continuous mediation model successfully established the overarching masking effect of psychological resilience, this aggregated approach inherently obscures nuanced interactions. To address this, the psychological network analysis was necessary to deconstruct these constructs at a micro-level, revealing how specific facets of wisdom act as active drivers of psychological burden. Building upon these mechanistic insights, our cluster analysis provided a necessary person-centered validation, demonstrating that the theoretical buffering effect observed in our models translates into real-world adaptation only when these protective traits concurrently manifest within the same individual. Together, these advanced analyses operate cohesively to robustly substantiate the central masking mechanism.

### Comparison with prior studies

Traditionally, wisdom is defined as an adaptive capacity that helps individuals overcome psychological barriers (Ardelt and Jeste, 2018). Existing research predominantly highlights its positive effects on mental health (Van Patten et al., 2019), such as alleviating mental disorders caused by childhood trauma among college students (Zhang et al., 2022). Similarly, psychological resilience is widely recognized as a key factor in actively coping with traumatic life events, with studies showing it reduces the risk of PLEs (Gloria and Steinhardt, 2016) and helps maintain psychological balance during disasters and crises (Nishi et al., 2016). Relevant studies have also pointed out that individuals with a high level of wisdom usually possess greater psychological resilience (Jeste et al., 2025). However, our finding that high wisdom entails corresponding psychological risks contradicts some previous research results. Wisdom is a complex, multi-dimensional construct, and excessively highlighting certain sub-dimensions may become a source of risk. While prosocial behavior is generally seen as a social lubricant (Pfattheicher et al., 2022), an emerging body of literature warns that it can cause psychological problems (Cameron et al., 2019) and act as a “double-edged sword” (Wang et al., 2025). Excessive empathic concern can lead to over-adaptation, increasing the risk of depression (Wang et al., 2025), and exerting effort to help others is accompanied by greater stress (Pronizius et al., 2024). Interestingly, studies have shown that doctors often exhibit significantly lower emotional empathy than the general population, which may serve as a necessary self-protection mechanism in special professional environments (Hojat et al., 2009).

### Mechanisms

To unpack the mechanisms driving this masking effect, we integrate the trait wisdom model, the stress-buffering hypothesis of resilience theory, and the psychosis continuum framework into a unified cognitive-emotional regulation model. From the perspective of the trait wisdom model, wisdom is not merely a static asset but an active process involving complex cognitive and emotional engagement. Our network analysis provides data-driven insights into this process: prosocial behaviors and decisiveness were central nodes directly connected to persecutory ideation (PI) and bizarre experiences (BE). Engaging in prosocial behavior—whether viewed from a consequentialist or intentionalist perspective (Pfattheicher et al., 2022) —requires individuals to place their own interests in a secondary position and necessitates rigorous risk assessment (Weissman, 1976). Furthermore, acute stress can reduce effortful prosocial behaviors (Forbes et al., 2024), and investing time or money into altruism causes significant cognitive burden (Cameron et al., 2019). Neurobiological studies further reveal that positive prosocial behaviors and negative risk-taking behaviors exhibit overlapping neural circuits (Telzer, 2015), which may explain why adolescents frequently engage in prosocial risk-taking behaviors (Do et al., 2017). This cognitive pressure may force a decisive shift towards impulsivity, given the significant correlation between stress exposure and impulsivity (Seldin et al., 2023). Crucially, our network analysis also revealed that resilience nodes exhibited high betweenness centrality. Structurally, these nodes act as critical hubs that intercept the pathways between wisdom-induced cognitive burden and the manifestation of PLEs.

Within the psychosis continuum framework, PLEs exist on a spectrum of severity. The intense cognitive burden, risk-taking, and impulsivity generated by effortful trait wisdom can act as a psychosocial stressor, potentially pushing individuals toward heightened subclinical vulnerability on this continuum. Crucially, the stress-buffering hypothesis derived from resilience theory provides the pivotal regulatory mechanism within this unified framework. Highly resilient individuals possess the internal adaptive resources to offset these cognitive and emotional risks (Fergus and Zimmerman, 2005) and effectively cope with psychological crises (Jeste et al., 2025). Therefore, the statistical masking effect we observed is theoretically explained by a dual-pathway mechanism: while the cognitive-emotional processing inherent in high wisdom increases baseline psychosocial stress, psychological resilience simultaneously acts as a powerful stress-buffer, absorbing this internal strain and mitigating the expression of positive PLEs.

### Implications

The intricate interplay among wisdom, resilience, and mental health holds significant clinical implications. Based on our findings, while high wisdom levels offer adaptive benefits, the pursuit of specific sub-dimensions without adequate psychological scaffolding may introduce subclinical vulnerabilities. To safely harness the benefits of wisdom, university mental health programs should implement targeted strategies derived from our empirical results: First, psychoeducational strategies must address the “double-edged sword” nature of wisdom. Our network analysis directly linking prosocial behaviors to positive symptoms suggests that counselors should train students to balance altruistic behaviors with healthy boundaries, thereby preventing empathy-induced cognitive burnout. Second, our mediation model highlights the critical necessity of resilience-based interventions. Because resilience acts as a vital masking variable that absorbs the cognitive costs of high wisdom, universities must proactively deploy psychosocial programs specifically designed to enhance an individual’s psychological buffering capacity against real-world adversities. Third, our findings underscore the importance of early screening for PLEs. Routine mental health assessments are particularly crucial for students exhibiting excessive prosocial risk-taking or high cognitive burdens, as our data indicate these traits may mask silent subclinical vulnerabilities. Finally, given potential cultural deviations, standardizing the definition of wisdom remains valuable for developing universally effective mental health frameworks.

## Limitations and future directions

All participants were from a single center, which may lead to participant bias. Additionally, a relatively high level of education may affect wisdom levels. Although the researchers used partial correlation to minimize the interference of additional factors on the results, they did not examine the impact of intelligence on wisdom levels. Previous research has suggested that intelligence is a necessary but insufficient condition for wisdom. Therefore, in future research, the researchers will focus on whether there are differences in wisdom, psychological resilience, and mental state among people with different intelligence levels, and the possible reasons behind these differences. At the same time, this study only included college students as the subject group, so the conclusion still needs to be validated in a wider population to ensure its credibility.

Several methodological limitations must be critically acknowledged. The cross-sectional design inherently precludes the establishment of causal relationships among wisdom, psychological resilience, and PLEs. Although theoretically grounded, our findings reflect statistical associations rather than deterministic pathways, necessitating future longitudinal or experimental designs to verify causal trajectories. Furthermore, relying on online convenience sampling from a single medical university introduces potential sampling bias. This approach may overrepresent students with higher digital literacy or greater willingness to participate, limiting the generalizability of our findings to the broader undergraduate population. Future studies should utilize multi-center stratified random sampling to enhance external validity. Additionally, the reliance on self-report measures is highly susceptible to social desirability and subjective recall biases, particularly when assessing socially esteemed constructs like wisdom and prosocial behavior. Incorporating multi-informant assessments or objective behavioral metrics in future research would help triangulate these constructs and minimize subjective bias. Lastly, without rigorous clinical diagnostic data from our non-clinical sample, investigating these dynamics within high-risk cohorts remains crucial to determine if this masking mechanism operates similarly at clinical thresholds.

## Conclusion

Based on our cross-sectional design and network analysis, the impact of wisdom on mental state is highly multi-faceted. Contrary to the traditional view of wisdom as a universally protective trait, we reveal that high levels of specific dimensions (e.g., prosociality and decisiveness) are associated with potential subclinical vulnerabilities due to hidden cognitive burdens. However, by theoretically integrating the stress-buffering hypothesis, our findings systematically demonstrate that psychological resilience acts as a critical masking variable. This resilient scaffolding effectively mitigates the negative psychosocial impacts of high wisdom. Scientifically, this underscores that the adaptive value of wisdom is inextricably linked to an individual’s emotion-regulation capacity. Finally, given potential deviations across cognitive levels and cultures, standardizing a broader, multi-dimensional definition of wisdom remains a crucial direction for future psychological research and clinical interventions.

## Data Availability

The original contributions presented in the study are included in the article/Supplementary material, further inquiries can be directed to the corresponding author/s.

## Footnotes

1
https://github.com/zhuyp626/SD-WISE-data-analysis.git

## References

[ref1] Ardelt M. Jeste D. V. (2018). Wisdom and hard times: the ameliorating effect of wisdom on the negative association between adverse life events and well-being. J. Gerontol. B Psychol. Sci. Soc. Sci. 73, gbw137–gbw1383. doi: 10.1093/geronb/gbw137, 28329810 PMC6178964

[ref2] Baltes P. B. Smith J. (1990). “Toward a psychology of wisdom and its ontogenesis,” in Wisdom: Its Nature, Origins, and Development, ed. Sternberg R. J. (Cambridge: Cambridge University Press), 87–120. doi: 10.1017/CBO9781139173704.006

[ref3] Baron R. M. Kenny D. A. (1986). The moderator-mediator variable distinction in social psychological research: conceptual, strategic, and statistical considerations. J. Pers. Soc. Psychol. 51, 1173–1182. doi: 10.1037//0022-3514.51.6.1173, 3806354

[ref4] Birren J. E. Svensson C. M. (2005). “Wisdom in history,” in A Handbook of Wisdom: Psychological Perspectives, eds. Jordan J. Sternberg R. (Cambridge: Cambridge University Press), 3–31. doi: 10.1017/CBO9780511610486.002

[ref5] Blondel V. D. Guillaume J.-L. Hendrickx J. M. de Kerchove C. Lambiotte R. (2008). Local leaders in random networks. Phys. Rev. E 77:036114. doi: 10.1103/PhysRevE.77.036114, 18517468

[ref6] Cameron C. D. Hutcherson C. A. Ferguson A. M. Scheffer J. A. Hadjiandreou E. Inzlicht M. (2019). Empathy is hard work: people choose to avoid empathy because of its cognitive costs. J. Exp. Psychol. Gen. 148, 962–976. doi: 10.1037/xge0000595, 30998038

[ref9013] Capra C. Kavanagh D. J. Hides L. Scott J. (2013). Brief screening for psychosis-like experiences. Schizophr. Res. 149, 104–107. doi: 10.1016/j.schres.2013.05.020 23830544

[ref7] Chen W. Wang X. Sun S. Liu Q. Guo Z. (2022). The relationship between neuroticism and mobile phone use among college students in love: the masking effect of self-emotional assessment. Front. Psychol. 13:942520. doi: 10.3389/fpsyg.2022.942520, 36186322 PMC9520978

[ref9015] Davydov D. M. Stewart R. Ritchie K. Chaudieu I. (2010). Resilience and mental health. Clin. Psychol. Rev. 30, 479–495. doi: 10.1016/j.cpr.2010.03.003 20395025

[ref8] Do K. T. Guassi Moreira J. F. Telzer E. H. (2017). But is helping you worth the risk? Defining prosocial risk taking in adolescence. Dev. Cogn. Neurosci. 25, 260–271. doi: 10.1016/j.dcn.2016.11.008, 28063823 PMC5461219

[ref9] Dong M. Weststrate N. M. Fournier M. A. (2023). Thirty years of psychological wisdom research: what we know about the correlates of an ancient concept. Perspect. Psychol. Sci. 18, 778–811. doi: 10.1177/17456916221114096, 36322834 PMC10336627

[ref10] Fairchild A. J. McDaniel H. L. (2017). Best (but oft-forgotten) practices: mediation analysis. Am. J. Clin. Nutr. 105, 1259–1271. doi: 10.3945/ajcn.117.152546, 28446497 PMC5445681

[ref11] Fergus S. Zimmerman M. A. (2005). Adolescent resilience: a framework for understanding healthy development in the face of risk. Annu. Rev. Public Health 26, 399–419. doi: 10.1146/annurev.publhealth.26.021304.144357, 15760295

[ref12] Forbes P. A. G. Aydogan G. Braunstein J. Todorova B. Wagner I. C. Lockwood P. L. . (2024). Acute stress reduces effortful prosocial behaviour. eLife 12:RP87271. doi: 10.7554/eLife.87271, 38180785 PMC10942768

[ref13] Gloria C. T. Steinhardt M. A. (2016). Relationships among positive emotions, coping, resilience and mental health. Stress. Health 32, 145–156. doi: 10.1002/smi.2589, 24962138

[ref14] Glück J. Scherpf A. (2022). Intelligence and wisdom: age-related differences and nonlinear relationships. Psychol. Aging 37, 649–666. doi: 10.1037/pag0000692, 35587418

[ref15] Hojat M. Vergare M. J. Maxwell K. Brainard G. Herrine S. K. Isenberg G. A. . (2009). The devil is in the third year: a longitudinal study of erosion of empathy in medical school. Acad. Med. 84, 1182–1191. doi: 10.1097/ACM.0b013e3181b17e55, 19707055

[ref16] Huth K. B. S. Haslbeck J. M. B. Keetelaar S. van Holst R. J. Marsman M. (2025). Statistical evidence in psychological networks. Nat. Hum. Behav. 10, 333–346. doi: 10.1038/s41562-025-02314-2, 41068490

[ref17] Jeste D. V. Alexopoulos G. S. Blazer D. G. Lavretsky H. Sachdev P. S. Reynolds C. F. (2025). Wisdom, resilience, and well-being in later life. Annu. Rev. Clin. Psychol. 21, 33–59. doi: 10.1146/annurev-clinpsy-081423-031855, 39621412

[ref18] Jiang Y. Zhao X. Zhu L. Liu J. S. Deng K. (2021). Total-effect test is superfluous for establishing complementary mediation. Stat. Sin. 31, 1961–1983. doi: 10.5705/ss.202019.0150

[ref19] Lee E. E. Bangen K. J. Avanzino J. A. Hou B. Ramsey M. Eglit G. . (2020). Outcomes of randomized clinical trials of interventions to enhance social, emotional, and spiritual components of wisdom: a systematic review and meta-analysis. JAMA Psychiatry 77, 925–935. doi: 10.1001/jamapsychiatry.2020.0821, 32401284 PMC7221873

[ref9009] Linscott R. J. van Os J. (2013). An updated and conservative systematic review and meta-analysis of epidemiological evidence on psychotic experiences in children and adults: on the pathway from proneness to persistence to dimensional expression across mental disorders. Psychol. Med. 43, 1133–1149. doi: 10.1017/S0033291712001626 22850401

[ref9010] Livet A. Navarri X. Potvin S. Conrod P. (2020). Cognitive biases in individuals with psychotic-like experiences: a systematic review and a meta-analysis. Schizophr. Res. 222, 10–22. doi: 10.1016/j.schres.2020.06.016 32595098

[ref9008] Lu D. Wang W. Qiu X. Qing Z. Lin X. Liu F. . (2020). The prevalence of confirmed childhood trauma and its’ impact on psychotic-like experiences in a sample of Chinese adolescents. Psychiatry Res. 287:112897. doi: 10.1016/j.psychres.2020.112897 32203750

[ref9011] Mao Y. Zhang N. Liu J. Zhu B. He R. Wang X. (2019). A systematic review of depression and anxiety in medical students in China. BMC Med. Educ. 19:327. doi: 10.1186/s12909-019-1744-2 31477124 PMC6721355

[ref20] Nishi D. Kawashima Y. Noguchi H. Usuki M. Yamashita A. Koido Y. . (2016). Resilience, post-traumatic growth, and work engagement among health care professionals after the great East Japan earthquake: a 4-year prospective follow-up study. J. Occup. Health 58, 347–353. doi: 10.1539/joh.16-0002-OA, 27265533 PMC5356942

[ref9012] Ong A. D. Bergeman C. S. Boker S. M. (2009). Resilience comes of age: defining features in later adulthood. J. Pers. 77, 1777–1804. doi: 10.1111/j.1467-6494.2009.00600.x 19807864 PMC2807734

[ref9014] Osborne K. J. Willroth E. C. DeVylder J. E. Mittal V. A. Hilimire M. R. (2017). Investigating the association between emotion regulation and distress in adults with psychotic-like experiences. Psychiatry Res. 256, 66–70. doi: 10.1016/j.psychres.2017.06.011 28624674

[ref21] Pfattheicher S. Nielsen Y. A. Thielmann I. (2022). Prosocial behavior and altruism: a review of concepts and definitions. Curr. Opin. Psychol. 44, 124–129. doi: 10.1016/j.copsyc.2021.08.021, 34627110

[ref22] Pronizius E. Forbes P. A. G. Feneberg A. C. Miculescu B. Nater U. M. Piperno G. . (2024). Everyday helping is associated with enhanced mood but greater stress when it is more effortful. Sci. Rep. 14:24120. doi: 10.1038/s41598-024-75261-z, 39407032 PMC11480085

[ref23] Qin X. Liu L. Yan Y. Guo X. Yang N. Li L. (2025). Smartphone addiction and sleep quality in the physical activity-anxiety link: a mediation-moderation model. Front. Public Health 13:1512812. doi: 10.3389/fpubh.2025.1512812, 40247873 PMC12003382

[ref9016] Scali J. Gandubert C. Ritchie K. Soulier M. Ancelin M. L. Chaudieu I. (2012). Measuring resilience in adult women using the 10-items Connor-Davidson resilience scale (CD-RISC). Role of trauma exposure and anxiety disorders. PLoS One 7:e39879. doi: 10.1371/journal.pone.0039879 22768152 PMC3387225

[ref24] Seldin K. Lengua L. J. King K. M. (2023). The relation between stress and impulsivity during the first year of college. J. Pers. 91, 1189–1206. doi: 10.1111/jopy.12792, 36377955

[ref9017] Sun M. Wang D. Jing L. Xi C. Dai L. Zhou L. (2020). Psychometric properties of the 15-item positive subscale of the community assessment of psychic experiences. Schizophr. Res. 222, 160–166. doi: 10.1016/j.schres.2020.06.003 32522467

[ref25] Telzer E. H. (2015). Dopaminergic reward sensitivity can promote adolescent health: a new perspective on the mechanism of ventral striatum activation. Dev. Cogn. Neurosci. 17, 57–67. doi: 10.1016/j.dcn.2015.10.010, 26708774 PMC4727991

[ref9018] Thomas M. L. Bangen K. J. Palmer B. W. Sirkin Martin A. Avanzino J. A. Depp C. A. . (2019). A new scale for assessing wisdom based on common domains and a neurobiological model: the San Diego wisdom scale (SD-WISE). J. Psychiatr. Res. 108, 40–47. doi: 10.1016/j.jpsychires.2017.09.005 28935171 PMC5843500

[ref9019] Treichler E. B. H. Palmer B. W. Wu T. C. Thomas M. L. Tu X. M. Daly R. . (2022). Women and men differ in relative strengths in wisdom profiles: a study of 659 adults across the lifespan. Front. Psychol. 12:769294. doi: 10.3389/fpsyg.2021.769294 35185678 PMC8850272

[ref9020] Troy A. S. Willroth E. C. Shallcross A. J. Giuliani N. R. Gross J. J. Mauss I. B. (2023). Psychological resilience: an affect-regulation framework. Annu. Rev. Psychol. 74, 547–576. doi: 10.1146/annurev-psych-020122-041854 36103999 PMC12009612

[ref26] Van Patten R. Lee E. E. Daly R. Twamley E. Tu X. M. Jeste D. V. (2019). Assessment of 3-dimensional wisdom in schizophrenia: associations with neuropsychological functions and physical and mental health. Schizophr. Res. 208, 360–369. doi: 10.1016/j.schres.2019.01.022, 30773419 PMC6788800

[ref9021] Villacura-Herrera C. Pérez J. Jones P. B. Núñez D. (2024). Internal consistency and temporal stability of the community assessment of psychic experiences (CAPE): a reliability generalization meta-analysis. Psychiatry Res. 338:115988. doi: 10.1016/j.psychres.2024.115988 38850889

[ref28] Wang R. Zhang X. Zhu L. Teng H. Zhang D. Qiu B. (2025). The double-edged sword effect of empathic concern on mental health and behavioral outcomes: the mediating role of excessive adaptation. Behav. Sci. 15:463. doi: 10.3390/bs15040463, 40282084 PMC12024002

[ref29] Weissman M. S. (1976). Decisiveness and psychological adjustment. J. Pers. Assess. 40, 403–412. doi: 10.1207/s15327752jpa4004_10, 957087

[ref9022] Worthy D. A. Gorlick M. A. Pacheco J. L. Schnyer D. M. Maddox W. T. (2011). With age comes wisdom: decision making in younger and older adults. Psychol. Sci. 22, 1375–1380. doi: 10.1177/0956797611420301 21960248 PMC3212636

[ref9023] Wu Z. Jiang Z. Wang Z. Ji Y. Wang F. Ross B. . (2022). Association between wisdom and psychotic-like experiences in the general population: a cross-sectional study. Front. Psych. 13:814242. doi: 10.3389/fpsyt.2022.814242 PMC9058059 35509888

[ref9003] Wu Z. P. Li C. F. Han L. Fu X. Z. Yan X. Y. Deng Q. . (2023). Reliability and validity of the Chinese version of San Diego wisdom scale(SD-WISE) in college students[J].Chinese. J. Clin. Psychol. 31, 1482–1486. doi: 10.16128/j.cnki.1005-3611.2023.06.036

[ref30] Ye Z. J. Wang Z. Y. Liang M. Z. Knobf T. Dixon J. She Y. . (2018). Reliability and validity of the Chinese version of the 10-item Connor-Davidson resilience scale in Chinese cancer patients. Chin. Gen. Pract. 21, 1839–1844. doi: 10.3969/j.issn.1007-9572.2018.00.107

[ref31] Zhang J. Liu Z. Long Y. Tao H. Ouyang X. Wu G. . (2022). Mediating role of impaired wisdom in the relation between childhood trauma and psychotic-like experiences in Chinese college students: a nationwide cross-sectional study. BMC Psychiatry 22:655. doi: 10.1186/s12888-022-04270-x, 36271351 PMC9587544

