# Supplementary material for: The mediating role of college student psychological resilience in the relationship between wisdom and psychotic-like experiences
Source: Front Psychol. 2026 May 19;17:1814754. doi: 10.3389/fpsyg.2026.1814754 (PMC13226621; doi:10.3389/fpsyg.2026.1814754)
Supplement: Supplementary file 1 [file Data_Sheet_1.docx]

**Supplementary Materials**


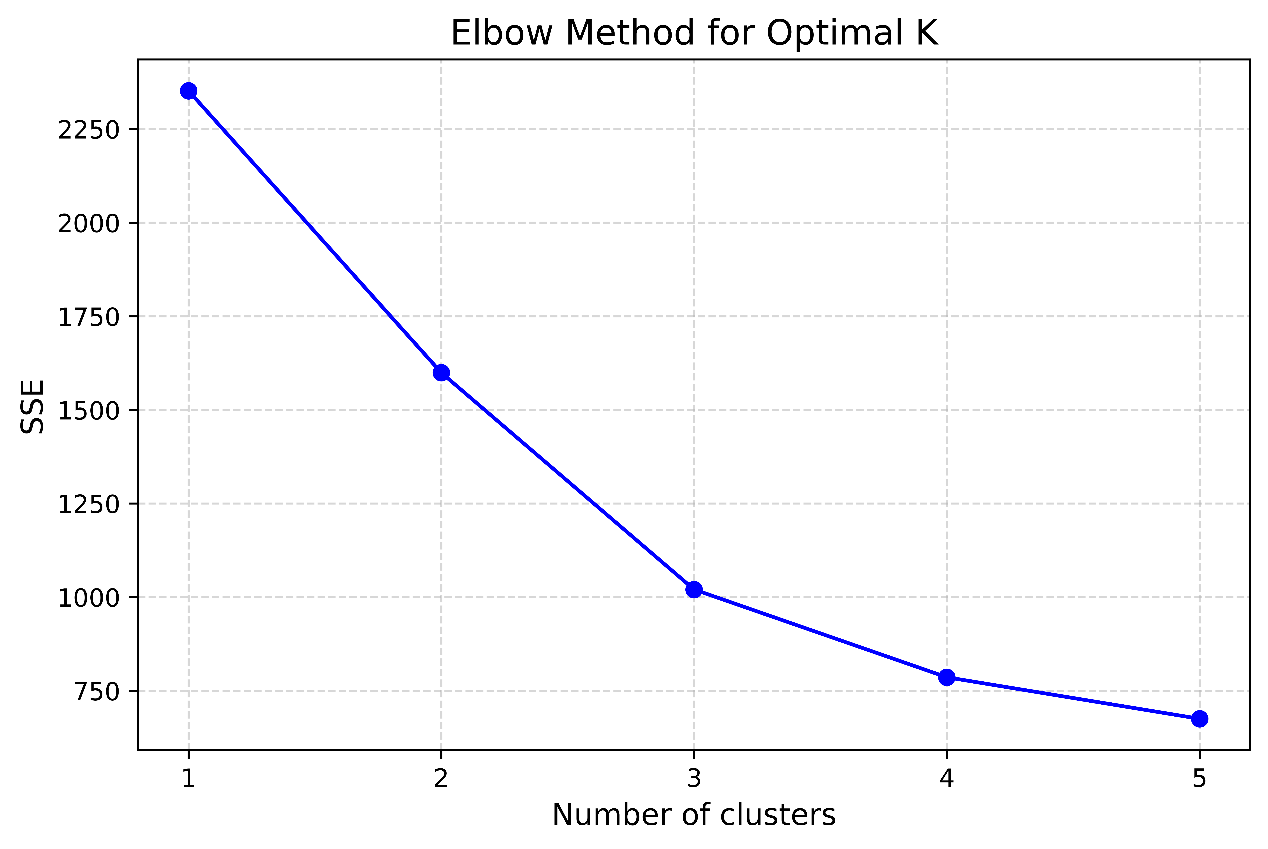


Supplementary Fig. 1 Elbow Method for Optimal K. We first used the elbow method to determine that the optimal number of clusters.


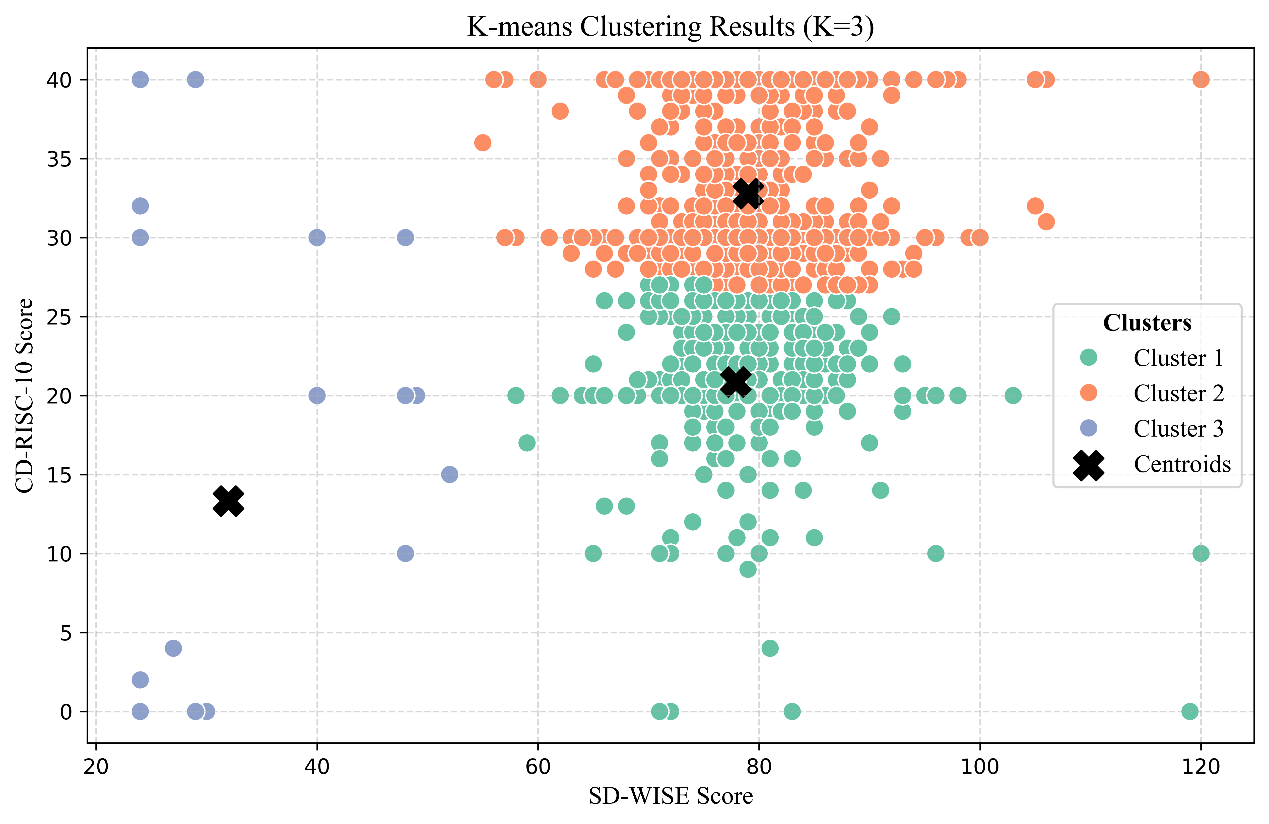


Supplementary Fig. 2 K-means Clustering Results (K = 3). The black cross marks indicate the cluster centroids. Green dots represent Cluster 1. Orange dots represent Cluster 2. Blue dots represent Cluster 3.


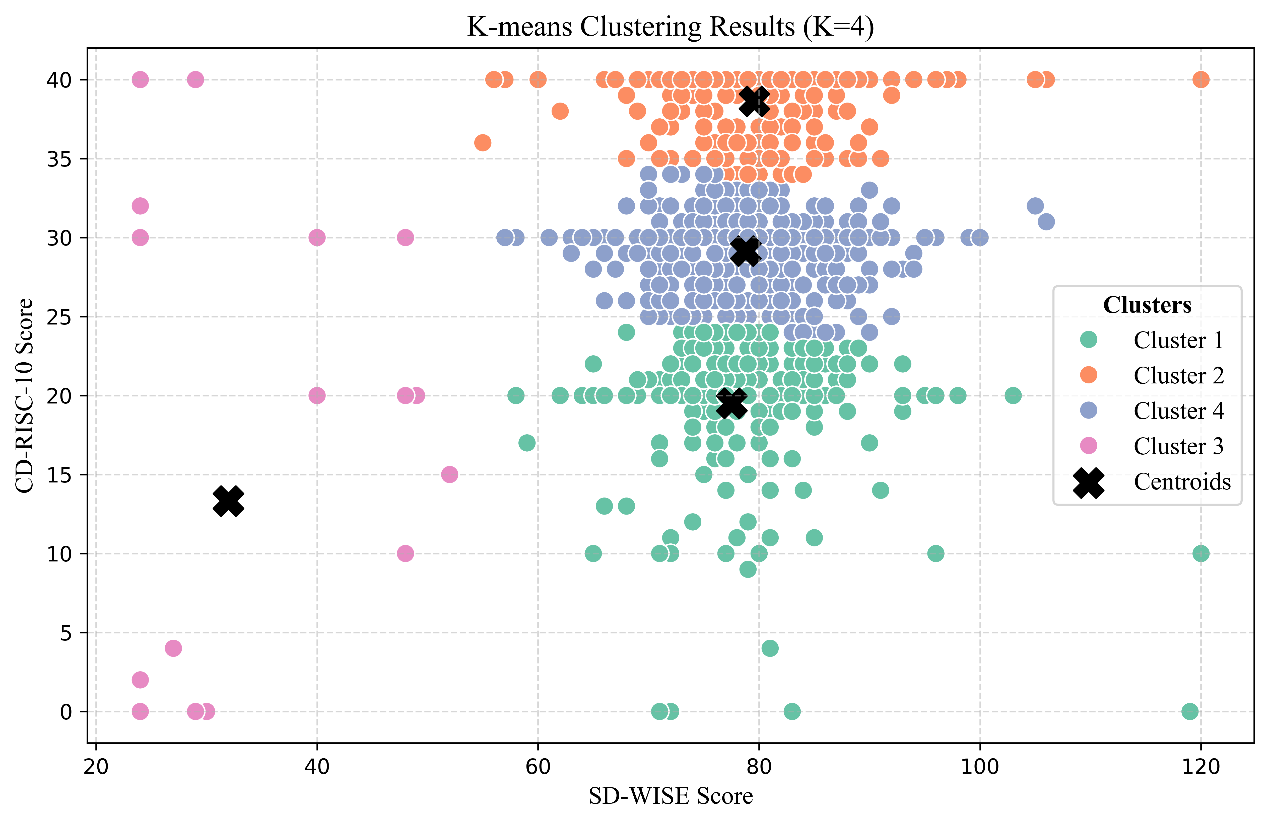


Supplementary Fig. 3 K-means Clustering Results (K = 4). The black cross marks indicate the cluster centroids. Green dots represent Cluster 1. Orange dots represent Cluster 2. Blue dots represent Cluster 3. Pinck dots represent Cluster 4.


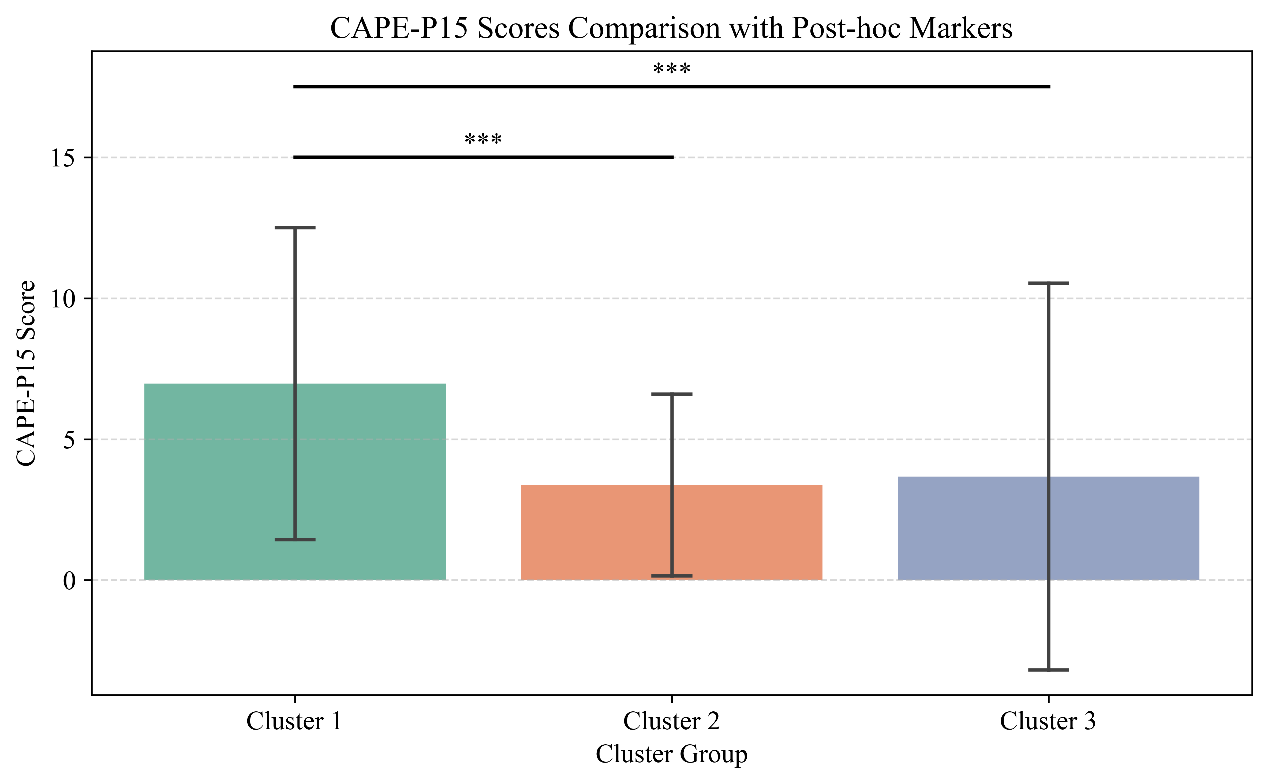


Supplementary Fig. 4 CAPE-P15 Scores Comparison Between Clusters. Results of the one - way ANOVA test on CAPE-P15 scores between the 3 clusters after k-mean cluster analysis. *** *p* < 0.001
